# Digital Deception: Survey Bots and the Ethics of Data Integrity

**DOI:** 10.1002/eahr.70017

**Published:** 2026-09-04

**Authors:** Whanyichukwu Frank‐Ito, Lori Ann Eldridge, Jennifer Matthews

**Affiliations:** ^1^ Student in the Department of Health and Human Performance at East Carolina University; ^2^ Assistant professor in the Department of Health and Human Performance at East Carolina University; ^3^ Professor in the Department of Health and Human Performance at East Carolina University

**Keywords:** survey bots, online recruitment, data integrity, artificial intelligence, ethical research, fraud mitigation, financial incentive, human research ethics

## Abstract

The rapid growth of artificial intelligence and automated systems has significantly impacted digital survey‐based research, particularly when recruitment is conducted via social media platforms. This case study details the erosion of data integrity in a 2025 follow‐up survey, originally designed to replicate a successful 2021 study. Despite implementing increasingly sophisticated antifraud mechanisms such as CAPTCHA, IP geo‐restrictions, and behavioral validation, survey bots repeatedly infiltrated the study, rendering the dataset unusable. This highlights a critical tension between broad digital accessibility and the vulnerability of online research to fraud. Moreover, the use of financial incentives to participate in research, while effective in increasing response rates, was found to amplify bot activity and raise ethical dilemmas surrounding compensation and data quality. As automated deception continues to evolve, researchers must navigate a complex landscape where conventional protections are no longer sufficient. The authors call for the urgent development of fieldwide standards, institutional support, and robust fraud prevention strategies to preserve the methodological and ethical integrity of digital research. Without such measures, the viability of online recruitment and the trustworthiness of the resulting data remain at serious risk.

In an era where nearly 80% of adults engage with social media platforms, online recruitment for survey‐based research has become both a practical and attractive strategy.^1^ The rise of artificial intelligence (AI) has further enhanced the feasibility of digital data collection, enabling a more efficient reach. For instance, one study found that AI‐enhanced surveys achieved a 94.2% completion rate, compared to 78.6% for traditional surveys, demonstrating AI's significant impact on improving participant engagement and data quality.^2^ However, this digital convenience brings new ethical and methodological challenges. One of the most notable among them is the infiltration of survey bots.^3^ In the context of online surveys, the term “survey bot” refers to a script or program that is written to complete a survey multiple times, often generating hundreds to thousands of responses with fraudulent information, with the goal of receiving multiple promised compensations. Survey bots can often be responsible for thousands of fraudulent responses within short periods of time. These automated agents can distort datasets in subtle yet meaningful ways, undermining the validity of research findings even when standard data cleaning procedures are applied.^4^, ^5^ As researchers increasingly turn to social media for participant recruitment, the presence of bots threatens the integrity of survey data, raising critical questions about the balance between accessibility and accuracy in digital research environments.

## CASE STUDY: THE EROSION OF DATA INTEGRITY IN A FOLLOW‐UP SURVEY

This case study details the methodological and ethical challenges encountered during our effort to replicate and expand upon a survey we originally conducted in 2021. The objective of the follow‐up was to re‐engage a demographically similar population using comparable recruitment strategies, specifically through social media platforms. However, the replication effort coincided with significant advancements in AI and the proliferation of automated survey bots, which ultimately compromised the integrity of the follow‐up data. The case illustrates how digital interference, particularly within online recruitment systems that offer financial incentives to participate, can directly compromise data integrity rather than merely distort research outcomes. When survey bots inject large volumes of fraudulent responses, the datasets themselves become ethically unusable, forcing researchers to confront difficult decisions about what, if any, information remains trustworthy. Beyond exposing the limitations of standard bot‐prevention methods, this case underscores an essential ethical responsibility in digital research: once compromised data is identified, researchers must determine how to transparently assess, manage, and report threats to data fidelity. Such dilemmas highlight the urgent need for more rigorous and adaptive data security and ethical‐governance frameworks to guide research teams when online collection is vulnerable to manipulation.

### Original survey context

The original survey was conducted in 2021 using social media platforms, primarily Facebook. To incentivize individuals to participate in the survey, they were informed that everyone who participated would be placed in a drawing that would result in 110 participants receiving a relatively generous financial payment of $75. Despite lacking any formal bot‐prevention mechanisms, the study yielded over 300 usable responses with no observable evidence of fraudulent activity or data anomalies. The data collection process was smooth, and the absence of digital tampering suggested that either bots had not yet become prevalent in survey‐based research or their technical capabilities at that time were insufficient to compromise the integrity of the study. The success of this initial effort informed the rationale for using a similar online recruitment strategy in the follow‐up study.

### Follow‐up survey and emergence of bot interference

In attempting to replicate the original study methodology, our follow‐up survey conducted in early 2025 was immediately faced with challenges due to bot interference. During the initial launch, no bot‐mitigation tools were used. Once Facebook advertisements promoting participant compensation of $30 were activated (of which 280 participants would be chosen from a lottery), the survey gathered over 2,000 responses within 24 hours, a figure that was vastly inconsistent with previous results. A preliminary data review using GeoIP analysis and other basic validation techniques confirmed that most responses were fraudulent, leading to an immediate stop in data collection.

In response, our second trial incorporated CAPTCHA verification to deter automated submissions. However, this measure proved ineffective, as bots continued to bypass the verification and inundate the survey with invalid entries, receiving hundreds of responses within the first hour of the survey's advertisement.

Our third trial introduced multiple layers of defense in addition to the previous prevention utilized, including IP‐based geo‐restrictions that limited participation to the state where the data was being collected and a targeted, open‐ended knowledge question specific to the county, which was the intended survey region. Yet even these additional safeguards were quickly circumvented. Within the first 50 failed submission attempts (approximately 15 minutes), bots had successfully understood that the survey utilized an IP‐based geo‐restriction and spoofed state‐based IP addresses and responded accurately to the location‐validation item, further illustrating the adaptability and sophistication of AI‐driven evasion techniques.

These repeated failures to preserve data integrity reveal the vulnerability of online surveys to digital deception and the inadequacy of current mitigation strategies. This case not only questions the continued viability of social media‐based recruitment in the era of AI but also underscores the ethical responsibility of researchers and institutions to proactively address threats to data authenticity in an increasingly automated digital landscape. This case revealed two main ethical issues: financial incentives to participate in research and methodological vulnerabilities.

## FINANCIAL INCENTIVES TO PARTICIPATE

While financial incentives accelerate enrollment in research studies, they also attract fraudulent actors who compromise data integrity and undermine ethical compensation practices. This emerging paradox challenges long‐standing assumptions about the feasibility of online research and demands careful reconsideration of the current methods of online recruitment when utilizing financial compensation.

Social media platforms have become a dominant avenue for participant recruitment in survey research, particularly when combined with financial incentives intended to increase response rates and sample diversity. In 2024, researchers from North Carolina found that offering a $15 incentive resulted in the quickest and most cost‐effective data collection, requiring only 17 hours and $338.64 in ad spending to achieve 142 non‐fraudulent survey completions. In contrast, a $5 incentive took 39 hours and $864.33 to achieve 148 completions, while a no‐incentive condition ran for 60 hours, spent nearly the full budget of $1,398.23, and achieved only 24 completions. Also, completion rates were higher in the incentivized conditions, with 93.4% for the $15 incentive and 89.8% for the $5 incentive, compared to 43.6% for the no‐incentive condition.^6^ However, financial incentives to participate in research make surveys prime targets for fraudulent activity, especially from increasingly sophisticated survey bots and coordinated fraud networks. This results in a troubling paradox: while incentives are designed to enhance participation, they simultaneously jeopardize the integrity of the dataset by attracting automated and deceptive responses. When individuals contribute their time and personal information with the expectation of compensation, researchers have a responsibility to honor that commitment. Bot interference can erode the funds allocated for participant payments, creating a situation where legitimate participants cannot be compensated. Failing to provide compensation, despite participants upholding their end of the research agreement, not only undermines trust in the research process but also violates a core ethical obligation.^7^

## RISE OF SURVEY BOTS

A growing body of evidence suggests that the prevalence of survey fraud is escalating. In recent assessments of online survey quality, researchers have documented a sharp decline in usable responses, from approximately 75% to as low as 10% in just a few years, largely due to undetected or poorly mitigated bot activity.^7^ This shift challenges the assumption that most respondents are genuine and places researchers in an ethical bind when survey administration is compromised. The underlying issue lies in the increasing difficulty of distinguishing between authentic participants and fraudulent actors. Bots can now bypass common security measures, mimic human behavior, and even spoof geo‐locations.^8^ In some cases, human fraudsters engage in hybrid schemes, manually completing eligibility

**The question is not whether online incentivized recruitment is still viable, but rather if researchers and their institutions are willing to adapt to the ever‐evolving threat of AI‐generated survey bots.**

screeners before deploying automated agents to finish the remainder of the survey.^7^ Determining legitimate human participants to receive compensation is nearly impossible. When the cost of manually vetting responses outweighs available resources, many researchers are left with no viable alternative but to classify the entire survey attempt as compromised. While the original intent of the incentive is to reward meaningful participation and facilitate data collection, this methodology becomes questionable when the resulting dataset is too compromised to analyze. In such cases, the foundational purpose of utilizing incentives is effectively nullified, rendering the use of incentives entirely ineffective. These developments call into question the long‐term viability of online recruitment for incentivized surveys. As deception methods continue to evolve, so do the ethical frameworks and technical safeguards that guide digital research. Without more advanced verification technologies and clearer policies regarding respondent compensation (i.e., consent) in cases of data compromise, researchers risk both ethical lapses and the erosion of public trust in survey‐based research. This, in turn, raises significant ethical concerns by placing the researcher in a position where they may unknowingly allocate or report the use of limited research resources, such as participant compensation funds, in ways that are inconsistent with ethical research stewardship. The increasing prevalence of fraudulent responses suggests a troubling trajectory, one in which online survey recruitment (with the assumption that incentives are being offered) may no longer be a financially reliable method for collecting data.

## CHALLENGES AND ETHICS RELATED TO DIGITAL RECRUITMENT

Researchers who collect data through online platforms bear the responsibility of safeguarding the methodological integrity of their studies. They are accountable for ensuring the integrity of research data, adhering to professional standards throughout the research process.^9^ However, the rapidly evolving nature of digital fraud presents a significant challenge to maintaining this standard. The ability to guarantee the reliability of survey responses has become increasingly uncertain, raising ethical concerns about the continued use of online recruitment when the authenticity of responses cannot be confidently verified. Researchers have noticed an increase in automated and fraudulent survey responses that jeopardize data validity; even traditional safeguards are often bypassed.^10^

Efforts to detect and prevent fraudulent survey activity have not kept pace with the increasingly sophisticated tactics used to manipulate online survey systems. Zhang et al. found that common antifraud techniques are often insufficient against sophisticated bots that can mimic human behavior and bypass standard security measures.^11^ Many platforms rely on standard security features, such as CAPTCHA or IP address filtering; however, these methods are increasingly ineffective.^12^ Fraudulent actors have developed tools and strategies that can mimic human behavior, bypass automated verification, and submit seemingly valid but ultimately deceptive responses. In one recent study, despite several preventative measures, many fraudulent respondents could provide survey responses that effectively mimicked legitimate respondents at first glance.^13^ These developments have made it increasingly difficult to ensure that data collected online accurately reflects the target population.

The burden of assessing the validity of survey data falls on the researcher. Yet, many investigators, particularly those in disciplines without advanced technical training, may lack the resources, institutional support, or methodological expertise to detect or manage fraudulent activity at the scale it now occurs. Researchers frequently encounter difficulties in identifying and counteracting fraudulent responses when recruiting participants online.^10^ When researchers are unable to differentiate between legitimate and illegitimate responses, the utility of the data becomes questionable, and the broader conclusions of the research may be invalidated.

This raises a critical question: can the continued use of online recruitment methods be ethically justified in light of these vulnerabilities? This question has no definitive answer, as there are many aspects to it that are ever‐changing as technology changes; however, there are a few questions that will withstand the test of time as technology continues to develop.

### By withholding payment to genuine human participants, would researchers be devaluing participants’ time and contributions?

While this question offers a different aspect on surveys and fraudulent responses in terms of understanding the use of humans and compensation via incentives, it is important to understand the use of incentives. Concerning surveys, the purpose of incentives is to reduce the potential for failing to enroll enough participants needed to obtain a sufficient number of survey responses for the research. Accounting for and identifying fraudulent responses, especially among survey bots, is nearly impossible. More often than not, survey bots and other forms of fraudulent responses slip through flagging and security measures. Thus, an argument about wasting human participants’ time on surveys becomes rational once it is understood that, in many cases, the differentiation between authentic humans and survey bots is an imaginary line. If researchers send money to respondents they think are human and it is later discovered that the money was sent to a survey bot, this means that research funds were improperly used as a result of a researchers’ lack of knowledge and preparation in differentiating human respondents from survey bots. This misuse of funds would occur because it is difficult to consistently differentiate valid from fraudulent responses. Thus, it makes sense to withhold finite resources such as research funds to compensate participants when a researcher cannot confidently distinguish between legitimate and fraudulent responses. Moreover, because research funds are limited, there is an ethical responsibility to ensure that these funds are not spent under conditions of uncertainty where payments may inadvertently reward bots rather than genuine participants.

To what extent does the acceptance of potentially fraudulent data in human subjects research erode public trust, misinform stakeholders, and constitute a form of research misconduct through negligent disregard for data integrity? The acceptance of fraudulent data poses profound ethical risks in human subjects research. Beyond undermining the scientific record, the publication of false or distorted findings can misdirect clinical practice, policy decisions, and resource allocation. This can ultimately affect the lives of the very individuals research is intended to benefit. When inaccurate data is used to inform real‐world actions, the consequences may include ineffective or harmful interventions. Safeguarding data integrity is therefore not only a methodological requirement but an ethical imperative central to responsible research. While researchers should understand the risks that come with conducting online surveys utilizing social media as the format of recruitment, it is important to consider whether they are responsible for publishing their data without being completely certain that the information they have posted is viable. Some may argue against publishing any research findings that are at risk for containing fraudulent responses. However, by removing the use of online surveys that utilize online recruitment, researchers lose the ease of access to multiple populations that comes with the wide reach of social media. Moving away from the use of social media while offering more reliable results delays a process that may take weeks via social media into a process that may take months or even years. This delay in data collection cannot be quantified in terms of fraudulent results, as fraudulent responses depend on the screening methods in place for a given survey; however, researchers can assume that data collected via methods outside of online recruitment will avoid fraudulent responses. While researchers cannot eliminate all fraudulent responses, they can reduce the risk of heavily falsified data by implementing stronger screening and verification methods. With such measures in place, the reach of social media can still be leveraged through a deliberate commitment to methodological rigor and data integrity.

### If researchers knowingly disseminate data that contains fraudulent responses, are they at risk of research misconduct?

To understand this question, research misconduct must be defined. Research misconduct is the “fabrication, falsification, or plagiarism in proposing, performing, or reviewing research, or reporting research results.”^14^ Instead of approaching the inclusion of fraudulent responses as a type of misconduct, the issue should instead be reframed as a limitation. Similar to large‐scale studies that have multiple processes of data analysis and cleaning, the use of fraudulent responses should not be approached as a problem that can be eliminated. Instead, fraudulent responses should be viewed as a component of a data set that are eliminated from the data set to the best of the researcher's ability, e.g., by removing all obvious cases that are typically flagged by intra‐survey detection. As researchers undergo the process of cleaning data, the presence of residual fraudulent responses is a methodological constraint, rather than an act of research misconduct. The threat of misconduct arises only when a researcher knowingly ignores clear evidence of fraudulent data that may misrepresent the quality of data collected. Therefore, a researcher's determinations about the inclusion of fraudulent responses should be approached as a matter of transparency rather than as a violation of the principles of responsible research if the researcher took a systematic approach to protecting the integrity of their data.

The convenience and reach of digital platforms to recruit survey participants remain appealing. As such, the increasing prevalence of compromised data calls for a reexamination of standard research practices. Without the development of more advanced detection tools and greater institutional support for secure data collection, the viability of online research may be jeopardized. Expecting researchers to weigh the benefits of rapid, large‐scale data collection against the risks of compromised findings means acknowledging that methodological rigor and ethical responsibility are fundamentally intertwined.

## MITIGATION STRATEGIES

Given the ethical risks outlined above, the challenge is not simply identifying the presence of fraudulent data but determining how researchers can ethically respond to the growing vulnerabilities inherent in online data collection. As digital recruitment becomes increasingly common and increasingly targeted by automated systems, researchers must adopt intentional strategies that protect data integrity while upholding the core principles of respect for persons, beneficence, and justice. Mitigation, then, is not merely a technical exercise; it is a fundamental ethical responsibility. The following section outlines practical and ethically grounded strategies for designing, implementing, and overseeing online survey research in ways that minimize fraud, safeguard participants’ rights, and ensure that the resulting data remain credible and fit for use in decision‐making that affects human lives.

### Financial incentives for human participants

Financial incentives have been used, and are often essential, to increase response rates and ensure equitable access to research opportunities. Yet the same incentives originally intended to achieve enrollment goals and acknowledge a person's time participating in research have created an ethical vulnerability.

How can researchers design and manage financial incentives in online surveys to reduce fraudulent participation while maintaining fairness and ensuring participant protection? A clear and transparent consent form can help mitigate ethical risk by setting expectations around eligibility, compensation conditions, and data validation procedures (i.e., what/if any, data will be used if bots are detected). For example, consent forms can explicitly state that compensation may be provided only after verification of response integrity or eligibility criteria, or that automated activity will render a response ineligible for payment. While such disclosures within the consent form may not eliminate fraud, they do strengthen ethical clarity by informing the participants of the steps needed to protect the research.

Researchers can also strengthen protections against paying fraudulent participants by incorporating an eligibility screener before the participants enter the full survey. Screeners inform participants during the consent process that eligibility will be evaluated using the brief screener; this further enhances transparency and ensures that compensation is provided only to those who meet the study criteria.

Other mitigation responses may include a multistep verification before payment is confirmed. This may include requiring human participants to provide validation of their identity and extensive documentation to prove it. Another method may be conditional payment, in which a human participant receives a nonmonetary item/token that they must exchange for the actual monetary payment after providing validation of their identity.

### Survey bots

Are the current mitigation strategies in place sufficient to allow the continuation of data collection via surveys using social media as the main source of recruitment? The growing prevalence of fraudulent survey responses has prompted the development of increasingly sophisticated methods for detection and prevention. While multiple systems exist to help safeguard data integrity, their effectiveness is variable, and none offer a definitive solution to ensure data integrity. Researchers must therefore approach online data collection with both caution and a strategic awareness of the limitations inherent in current technologies.

Among the most commonly employed safeguards are CAPTCHA protocols and challenge‐response tests, which aim to distinguish human users from automated systems. In one study, over 75% of survey entries were identified as fraudulent during data screening.^15^ Following the implementation of CAPTCHA at the entry point of the survey, no further evidence of automated submissions was detected. These results suggest that, when used early in the recruitment process, CAPTCHA can act as an effective first‐line defense.^15^ However, this measure alone is insufficient against more advanced forms of fraudulent behavior.

Additional layers of protection often include digital fingerprinting and Internet Protocol (IP) monitoring. These tools provide contextual information about the device and network associated with a given response, such as geo‐location, internet service provider, and usage type. While IP monitoring can flag suspicious patterns, such as multiple responses originating from a single address, its reliability is undermined by the use of proxy servers, virtual private networks, and other tools that obscure true user identity.^16^

In response to these limitations, researchers have begun to adopt behavioral analytics as part of their fraud detection strategies. These systems examine variables such as mouse movements, time between clicks, page dwell time, and consistency across responses to identify patterns that may indicate manipulation. Yet, as detection technologies have advanced, so have the methods used to bypass them. Fraudulent actors now employ techniques that mimic human behavior, including randomized delays, nonlinear navigation, scrolling activity, and deliberately inconsistent click patterns. This deliberate mimicry blurs the line between authentic and falsified behavior, weakening the reliability of behavior‐based detection models.

One of the most important mitigation strategies in deterring survey bots, as mentioned before, involves the use of bot screening systems. However, the dynamic between prevention systems and those who seek to undermine them reflects an ongoing pattern of adaptation and counter‐adaptation. As detection tools become more complex, fraudulent tactics evolve in response. This reality places a significant burden on researchers to remain informed about emerging threats and continuously evaluate the integrity of their data. It also underscores the need for institutions and survey platforms to invest in comprehensive security frameworks that integrate multiple layers of protection rather than relying on any single solution.

While current best practices can reduce the risk of compromised data, they cannot eliminate it entirely. Researchers must therefore balance efficiency in recruitment with vigilance in validation. Recognizing that methodological soundness depends not only on the quality of the data collected but also on the credibility of the processes used to secure it.

One clear implication is that data quality planning now must be a core component of study design. Researchers must assume from the outset that some portion of online responses will be fraudulent and build in safeguards accordingly.^8^ This may involve routinely including validation questions, randomizing survey flows, and incorporating real‐time fraud checks into the survey logic. Such measures add complexity to study design but are increasingly necessary to prevent bots from invalidating results. There is also a push for shorter recruitment windows and more controlled distribution of survey links (for example, using single‐use invitation codes) to limit exposure to bot networks.

Quality checks are necessary. However, if they're too strict, they can incorrectly exclude real participants, presenting a trade‐off for researchers. Determining the correct balance of screening questions is important to ensure that a survey filters out bots while also protecting the validity of real participants. Researchers must calibrate these defenses carefully: overly stringent checks can exclude genuine participants, harm sample quality, and potentially affect future studies. It is increasingly viewed as unethical to knowingly rely on data that could be heavily fabricated by bots, given the risk of false findings and wasted resources. Doing so implicates not only the researcher, but survey research in all fields of study. A researcher who knowingly engages in this type of misconduct opens the door to other questions about the work they reported. Research institutions and institutional review boards (IRBs) are increasingly requiring researchers to address data integrity risks in their protocols. And some IRBs require researchers to submit a plan for detecting and handling bogus cases, similar to requiring researchers to submit a plan to protect human participants’ privacy.

Yet, guidelines have lagged behind practice. Calls have been made to develop fieldwide standards or checklists that explicitly cover fraud detection and prevention measures, analogous to existing policies for reporting online research (e.g., Checklist for Reporting Results of Internet E‐Surveys). In the coming years, it is likely that professional organizations (such as the Association of Internet Researchers, or the American Association of Public Opinion Research) will release more explicit guidance on acceptable antifraud methods, disclosure, and fair compensation policies when fraud is suspected.^7^ The principle of transparency will need reinterpretation: researchers must be honest about their data cleaning criteria without empowering malicious actors. Maintaining trust is paramount—both the trust of participants that they will be treated fairly and the trust of society that research findings are based on responses from humans. Navigating this will likely become a formalized part of research ethics training as digital data collection continues to grow.

## CONCLUSION

Is incentive‐based data collection through online social media recruitment still viable, given the recent developments in survey bots and AI? While there is no definitive answer, it is clear that online methods remain one of the easiest forms for recruitment, as reaching large populations has never been simpler. The challenge in attempting to reach mass populations for data collection is the inability to distinguish between well‐intentioned populations and those with malicious intent. Consequently, data often contains a mix of legitimate responses and deceptive or fabricated entries.

While some may argue that the integrity of data collection lies with the researcher, technological developments make the process of legitimizing data unreasonably difficult and inconsistent. Yet survey bots and survey bot detection are constant cat‐and‐mouse games. Prevention and detection will always fall behind in development, putting researchers in a difficult position. Others may argue that ensuring ethical data collection lies with institutions and policies that enforce proactive measures to identify survey bots in the hopes of mitigating (if not preventing) collection of fraudulent data. Ideally, both institutions and IRBs should require researchers to submit and follow fraud mitigation protocols as mandatory components of study design.

In conclusion, the question is not whether online incentivized recruitment is still viable, but rather if researchers and their institutions are willing to adapt to the ever‐evolving threat of AI‐generated survey bots. Researchers will continue to bear the responsibility of maintaining ethical research practices to protect data integrity, retaining the trust of participants, and ensuring that the integrity of the data is safeguarded. If recruitment, detection, and prevention systems evolve alongside advancements in survey bots, then social media can be a powerful tool for researchers. However, if adaptation fails to keep pace, reliance on these methods will likely result in more harm than insight, producing data that is not only ethically questionable but also scientifically unusable.

## ACKNOWLEDGMENTS

Research reported in this publication was supported by the Centers for Disease Control and Prevention (CDC) under award number NH28CE003320. The content is solely the responsibility of the authors and does not necessarily represent the official views of the CDC. The authors thank the Pitt County Coalition on Substance Use.
